# Genetic diversity of submergence stress response in cytoplasms of the *Triticum-Aegilops* complex

**DOI:** 10.1038/s41598-018-34682-3

**Published:** 2018-11-02

**Authors:** Shotaro Takenaka, Ryohei Yamamoto, Chiharu Nakamura

**Affiliations:** Department of Plant Life Science, Faculty of Agriculture, Ryukoku University, 1-5 Yokotani, Ohe-cho, Seta, Otsu, 520–2194 Japan

## Abstract

Genetic diversity in cytoplasmic and nuclear genomes and their interaction affecting adaptive traits is an attractive research subject in plants. We addressed submergence stress response of wheat that has become increasingly important but remained largely uninvestigated. Our primary aim was to disclose cytoplasmic diversity using nucleus-cytoplasm (NC) hybrids possessing a series of heterologous cytoplasms in a common nuclear background. Effects of submergence on seedling emergence and growth from imbibed seeds were studied and compared with euplasmic lines. Marked phenotypic variabilities were observed among both lines, demonstrating divergent cytoplasmic and nuclear effects on submergence response. NC hybrids with cytoplasm of *Aegilops mutica* showed a less inhibition, indicative of their positive contribution to submergence tolerance, whereas cytoplasms of *Aegilops umbellulata* and related species caused a greater inhibition. Superoxide dismutase (SOD) activity showed a marked increase accompanied by retardation of seedling growth in a susceptible NC hybrid. The observation suggested that the elevated SOD activity was resulted from a high level of reactive oxygen species accumulated and remained in susceptible seedlings. Taken together, our results point to the usefulness of NC hybrids in further studies needed to clarify molecular mechanisms underlying the nucleus-cytoplasm interaction regulating submergence stress response in wheat.

## Introduction

Plants possess three interacting and coevolving genomes in three intracellular organelles, *i.e*. nucleus, mitochondria and chloroplasts. A vast majority of genetic information is stored in the nuclear genomes, whereas small numbers of protein-coding as well as non-coding genes reside in the two cytoplasmic genomes. Although small in size, owing to their essential roles in energy metabolisms, unique evolution through endosymbiosis and complex functional interactions with nuclear genomes, cytoplasmic genomes have provided researchers with many attractive subjects in plant science^1–5^. In contrast to nuclear genomes exhibiting Mendelian inheritance, cytoplasmic genomes compartmentalized in mitochondria and chloroplasts are generally inherited through maternal lineage in a majority of plant species. Taking advantage of this characteristic unilateral mode of inheritance, cytoplasms of related genera and species can be combined with given nuclei through repeated substitution backcrosses with recurrent paternal parents. To study intergeneric and interspecific diversity among cytoplasms in *Triticum* (wheat) and *Aegilops* (goatgrass) species, Kihara (1951) produced nucleus-cytoplasm substitution lines or nucleus-cytoplasm hybrids (hereafter abbreviated as NC hybrids, synonymous with alloplasmic lines and cytolines) and demonstrated that cytoplasm of *Ae. caudata* induced male sterility when it was introduced into common wheat (*T. aestivum*)^6^. This was the first demonstration of cytoplasmic male sterility in wheat. Ever since, a number of NC hybrids have been produced and utilized as valuable experimental materials in studying phylogeny and evolution of the tribe Triticeae. Particularly in the *Triticum-Aegilops* complex that comprises a series of diploid, allotetraploid and allohexaploid species evolved through hybridization followed by amphidiploidization between ancestral species^7^, a collection of 552 lines of NC hybrids have been produced by combining 12 different nuclear genomes of common wheat with 46 distinct cytoplasmic genomes covering all representative cytoplasms in the complex^8^. Based on extensive and systematic studies of cytoplasmic effects on fourteen vegetative and seven reproductive traits exhibited in these NC hybrids together with their chloroplast and mitochondrial DNA variations, cytoplasms of the *Triticum-Aegilops* complex were classified into 18 major types plus five subtypes^9,10^. NC hybrids thus contributed to establish the evolutional history in *Triticum* and *Aegilops* and greatly increased our knowledge of genetic architecture of both nuclear and cytoplasmic genomes^10^.

Studies of interspecific and intergeneric diversity in cytoplasms and nucleus-cytoplasm interactions have been extended to various phenotypes including male-sterility^11–13^, growth vigor^11^, viability^14^, inter-crossability and speciation^15,16^, photosynthesis and respiration^17,18^, transcriptomes and metabolomes^19^, and other agronomically important traits^20–22^. In such studies, hexaploid and tetraploid NC hybrids with cytoplasms of *Triticum*, *Aegilops*, *Agropyron, Haynaldia* and a wild relative of barley *Hordeum chilense* have been used. In maize also, NC hybrids with cytoplasms from teosinte including distantly related *Zea* species exhibited differences in morphological, physiological and developmental traits^23^. In the last decade, studies of a model plant species, *Arabidopsis thaliana*, have demonstrated intraspecific diversity of cytoplasms and nucleus-cytoplasm interactions both affecting a wide range of plant phenotypes. Studies using reciprocal F_2_ families or recombinant inbred lines showed variations in cytoplasmic genomes and cytonuclear interactions (synonymous with nucleus-cytoplasm interactions) that greatly affected natural variations in germination capacity and metabolomes^24,25^. Intraspecific cytonuclear interactions affecting various adaptive phenotypes in the fields have also been reported in a study using 56 cytolines produced by complete diallele crosses among eight natural *Arabidopsis* accessions^26^.

As above, inter- and intraspecific and intergeneric diversity of cytoplasms and nucleus-cytoplasm interactions affecting various phenotypes have been well investigated and documented in plants. However, contribution and role of cytoplasms in regulating complex adaptive traits, particularly responses to adverse environmental stresses in cereal crops, remain largely uninvestigated, although cytoplasmic genomes and nucleus-cytoplasm genome interaction have been known to play important roles in stress tolerance, signaling and adaptation in plants^27–31^. Submergence (complete inundation) and waterlogging (saturation of soil with water) caused by floods and heavy rainfalls have become serious threats in wheat production due to global climate changes^32–38^. Submergence together with waterlogging were reported to cause 15–20% yield reduction of wheat in the world, causing ever-increasing loss particularly in Asian countries where rice-wheat rotation is in a wide practice^33^. However, as compared with semiaquatic rice, our current knowledge of submergence/waterlogging stress response of wheat is quite limited. Because of the importance of this trait in rice, a large volume of research has been conducted and advanced our understanding of genetic, physiological, morphological and molecular mechanisms controlling the trait^35,36,39–42^. Two major strategies, i.e., “quiescent strategy” and “elongation or escape strategy”, have been known to be responsible for controlling submergence tolerance in rice. *SUB1A*, a gene encoding an ethylene responsive transcription factor (AP2/ERF), plays a key role in the operation of “quiescent strategy” by repressing cellular gibberellin levels^39^. By contrast, *SNORKEL 1, 2*, also encoding ethylene responsive transcription factors, play a key role in “elongation strategy” in deep-water or floating rice by enhancing gibberellin responses to induce rapid internode elongation^40^.

We focused on submergence stress response in wheat and initiated a study of cytoplasmic genetic diversity affecting seedling emergence and growth after subjecting imbibed seeds to submergence stress. To do this, we used a series of NC hybrids possessing heterologous cytoplasms derived from the *Triticum-Aegilops* complex combined with a common nucleus of a paternal donor parent, and compared their response to that of wheat accessions covering a wide range of genetic diversity. Also, we compared superoxide dismutase activity as a major antioxidant enzyme among a nuclear donor and tolerant and susceptible NC hybrids and discussed a possible involvement of cytoplasmic substitution in redox balance affecting submergence stress response. We herein report results providing the first experimental evidence for a large genetic diversity in both cytoplasmic and nuclear genomes affecting submergence stress response in wheat. Also, we point to the invaluable usefulness of NC hybrids as materials in further studies to elucidate molecular mechanisms underlying nucleus-cytoplasm interactions that regulate submergence stress response in wheat.

## Results

### Sensitivity of growing seedlings and imbibed seeds to submergence stress in a nuclear donor cultivar CS

**Figure 1:**
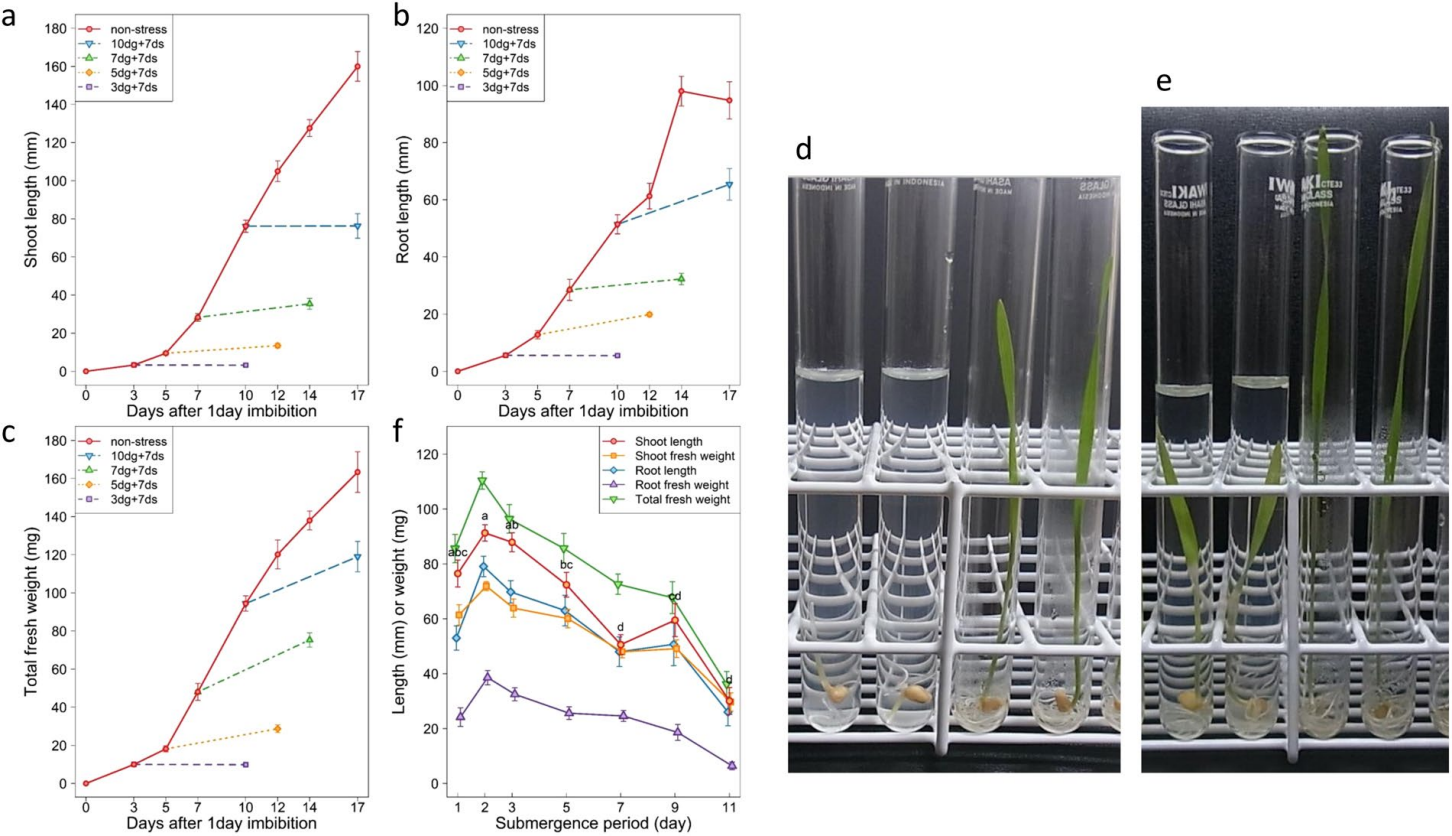
Inhibition of seedling growth by submergence imposed on seedlings and imbibed seeds. (**a**) Shoot length, (**b**) root length, and (**c**) total seedling fresh weight were measured at the indicated time points after submergence imposed on growing CS seedlings. Labels in the upper left indicate incubation conditions, e.g. 10dg + 7ds (10-day-old seedlings grown under non-submergence were submerged for 7 days). Bar represents mean ± SE. Photos of seedlings grown with and without submergence are shown in the followings. (**d**) Two on the right represent seedlings grown for 10 days without submergence (10dg) and two on the left seedlings submerged for 7 days after 3 days growth (3dg + 7ds). (**e**) Two on the right represent seedlings grown for 14 days without submergence (14dg) and two on the left seedlings submerged for 7 days after 7-days growth (7dg + 7ds). (**f**) Shoot length and fresh weight, root length and fresh weight, and total fresh weight were measured after submergence stress imposed on seeds during imbibition for 1 to 11 days followed by 10 days growth under de-submergence. Labels in the upper right indicate the five measured values. Bar represents mean ± SE. Mean comparisons of shoot length at different time points was made by Tukey’s test and shown at the 5% significance level.

Test tube bioassay developed for rice^43^ was adopted with modifications for wheat. Before studying cytoplasmic effects, we examined suitability of the bioassay method for evaluation of submergence stress response in wheat using a nuclear donor of standard common wheat cultivar Chinese Spring (abbreviated as CS). A time course of seedling growth without submergence was first studied using seeds imbibed for 1 day. Seed germination was 100% and seedlings continued to grow until the end of the incubation period in test tubes (Fig. 1a–c). Shoot length (length of either the first leaf or coleoptile depending on growth stage) and total seedling fresh weight continuously increased, whereas root length reached a plateau after 14 days of incubation, indicating that root growth was restricted during the later stages even without submergence stress under our bioassay conditions. Sensitivity of growing seedlings to submergence stress was then studied by adding deionized water to fully cover 3-, 5-, 7- and 10-day-old seedlings and keeping them submerged for additional 7 days. Submergence stress thus imposed on growing seedlings markedly suppressed further growth, showing that wheat seedlings were highly sensitive to complete submergence (Fig. 1a–c). Magnitude of inhibition was high, irrespective of time when the stress was administered. Photos of representative seedlings under different incubation conditions were shown in Fig. 1d,e. It was noted that under submergence the first leaves became necrotic at the junction of and above the top of coleoptile (Fig. 1e), showing that strong damage occurred in this region of the first leaves.

Sensitivity of seeds to prolonged submergence during imbibition was next studied by continuously exposing seeds to submergence for 1 to 11 days. Submerged seeds were then incubated under de-submergence conditions for additional 10 days for germination and subsequent seedling growth. Seed germination rate was 100% until 3–5 days of seed submergence and then declined steadily to 60% at the end of the 11^th^ day. Seedling growth assessed by all five traits (shoot length and fresh weight, root length and fresh weight, and total seedling fresh weight) reached peaks after 1 to 3 days of submergence and thereafter declined gradually over time (Fig. 1f). The result showed that an optimum period of seed imbibition was about 2 days under the assay conditions, and that the prolonged period of submergence gave strong stress on imbibed seeds and inhibited subsequent germination and seedling growth.

### Cytoplasmic diversity in NC hybrids assessed by seedling growth with and without submergence

**Figure 2:**
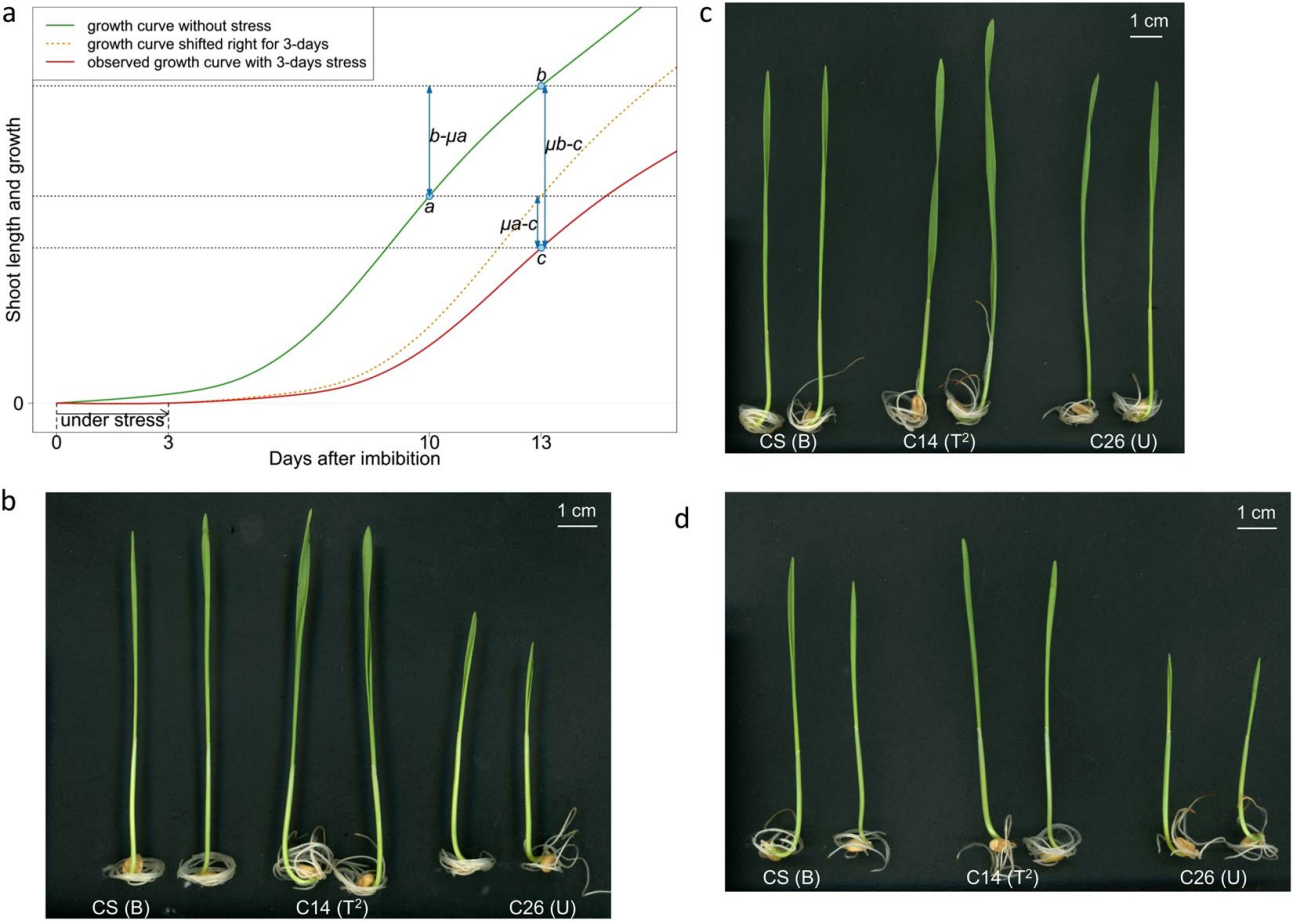
Experimental schemes for assessing seeding growth with and without submergence stress imposed on imbibed seeds and photos of representative seedlings. (**a**) Growth curves of shoot length (SL) without (green line) and with submergence stress (red line) after 2 days of imbibition. A broken orange curve represents a putative growth curve by shifting the green curve toward right for 3-days, assuming that stress caused only a delay of growth after de-submergence. Three variables (**a**–**c**) were measured at the indicated time points, and six derivative variables were calculated using the measured variables. *Variable a*: SL without submergence measured at the 10^th^ day (2di + 10dg); *b*: SL without submergence at the 13^th^ day (2di + 13dg); *c*: SL with 3-days submergence at the 13^th^ day (2di + 3ds + 10dg); *b-μa*: growth increment between the 10^th^ and the 13^th^ day; *μa-c*: growth inhibition by submergence compared with *μa*; *μb-c*: growth inhibition by submergence compared with *μb*; *(b-μa)/μa*: relative rate of growth increment for 3 days between the 10^th^ and the 13^th^ day; *(μa-c)/μa:* relative rate of growth inhibition by submergence compared with *μa*; *(μb-c)/μb*: relative rate of growth inhibition compared with *μb*. Photos of representative seedlings incubated under the three experimental schemes are shown in (**b**) 2di + 10dg, (**c**) 2di + 13dg and (**d**) 2di + 3ds + 10dg. From left to right, CS, C14 with T^2^ cytoplasm of *Ae. mutica* and C26 with U cytoplasm of *Ae. triuncialis*.

We studied effects of cytoplasmic genome substitution on seed germination and seedling growth after treating imbibed seeds with and without submergence using 37 lines of NC hybrids, each possessing one of 24 distinct cytoplasm types derived from the *Triticum-Aegilops* complex (Table 1). Three bioassay schemes were adopted for assessing the sensitivity to submergence (Fig. 2a). Submergence stress was imposed on 2 day-imbibed seeds by keeping them immersed for additional 3 days in deionized water and after releasing the stress they were incubated for 10 days under de-submergence (2di + 3ds + 10dg). Imbibed seeds were also incubated for 10 and 13 days without stress (2di + 10dg and 2di + 13dg, respectively). Three measurable variables *a, b* and *c* based on the five traits were used for assessing seedling growth. Variables *a* and *b* represented growth under the condition of 2di + 10dg and 2di + 13dg, respectively, and variable *c* represented growth under the condition of 2di + 3ds + 10dg. Six derivative variables were calculated in the following manners. Variable *b–μa*: 3-day growth increment between 2di + 13dg and 2di + 10dg. *(b-μa)/μa*: relative rate of growth increment compared with mean of *a*. *μa-c* and *μb-c*: growth inhibition by 3-day submergence compared with mean of *a* and *b*, respectively. *(μa-c)/μa* and *(μb-c)/μb*: relative rate of growth inhibition by 3-day submergence compared with mean of *a* and *b*, respectively.

**Table 1:** List of 37 NC hybrids and 12 hexaploid wheat lines including a nuclear donor CS used in the study.

Code	Cytoplasm donor	Genome	Plasmon
C01^mf^	*T. boeoticum* ^a^ *aegilopoides*	A	A
C02*	*Ae. caudata polyathera*	C	C
C03	*Ae. umbellulata*	U	U
C04	*Ae. squarrosa* ^b^ *typica*	D	D
C05*	*Ae. comosa* ^c^ *thessalica*	M	M
C07	*Ae. uniaristata*	N	N
C08	*Ae. speltoides ligustica*	S	S
C10	*Ae. sharonensis*	S^l^	S^l^
C11	*T. aestivum* ‘Panjamo’	BAD	B
C12	*Ae. bicornis*	S^b^	S^b^
C13	*Ae. mutica* ^d^	T	T
C14*	*Ae. mutica* ^d^	T	T^2^
C17	*Ae. speltoides aucheri* ^*e*^	S	S
C18	*Ae. searsii*	S^s^	S^v^
C19	*Ae. squarrosa* ^b^ *anathera*	D	D
C20	*Ae. longissima*	S^l^	S^l’^
C21	*T. dicoccoides* ^f^ *spontaneonigum*	BA	B
C22	*T. dicoccum*^g^ ‘Vernal'	BA	B
C26	*Ae. triuncialis*	UC	U
C28	*Ae. cylindrica*	DC	D’
C29	*Ae. biuncialis*	UM	U
C30	*Ae. columnaris*	UM	U’
C31	*Ae. ovata* ^h^	MU	M°
C32	*Ae. triaristata* ^i^	UM	U
C33	*Ae. kotschyi*	SU	S^v^
C34	*Ae. variabilis* ^j^	SU	S^v^
C35	*Ae. crassa*	DM	D^2^
C36	*Ae. ventricosa*	DN	D
C37	*Ae. biuncialis macrochaeta*	UM	U
C38	*Ae. triuncialis*	CU	C’
C39	*Ae. kotschyi*	SU	S^v^
C53	*Ae. juvenalis*	DMU	D^2^
C54	*Ae. triaristata* ^i^	UMN	U
C55	*Ae. crassa*	DMD	D^2^
C56	*Ae. vavilovii*	DMS	D^2^
C57	*Ae. triaristata* ^i^ *recta*	UMN	U
C58	*T. aestivum tibetanum*	BAD	B
Code	Hexaploid wheat	Genome	Plasmon
Tve	*T. aestivum* var. *erythrosperumum*	ABD	B
P168	*T. aestivum* strain P168	ABD	B
CS	*T. aestivum* cv. Chinese Spring	ABD	B
N26	*T. aestivum* cv. Norin 26	ABD	B
Slm	*T. aestivum* strain Salmon	ABD	B
JF	*T. aestivum* cv. Jones Fife	ABD	B
SK	*T. aestivum* cv. Selkirk	ABD	B
S615	*T. aestivum* cv. S-615	ABD	B
Sphr	*T. sphaerococcum* var. *rotundatum*	ABD	B
Cmp	*T. compactum* var. *humboldtii*	ABD	B
Splt	*T. spelta* var. *duhamelianum*	ABD	B
Mch	*T. macha* var. *subletschumicum*	ABD	B

Synonym: ^a^*T. monococcum* ssp. *aegilopoides*, ^b^*Ae. tauschii*, ^c^*Ae. comosa* var. *comosa*, ^d^*Ambylopyrum muticum*, ^e^*Ae. speltoides* ssp. *speltoides*, ^f^*T. turgidum* ssp. *dicoccoides*, ^g^*T. turgidum* ssp. *dicoccum*, ^h^*Ae. geniculate*, ^i^*Ae. neglecta*, ^j^*Ae. peregrine*. *Male-sterile lines. C01^mf^ shows a partial self-fertility with low plant vigor and is most likely a spontaneous revertant of line C01 with A-type cytoplasm from *T. boeoticum* (unpublished). Number of substitution backcrosses varied from 12 (for C13) to 35 (for C02) in all NC hybrids, except for 7 for C39 and 8 for C20. Table was modified after Tsunewaki (2009).

**Figure 3:**
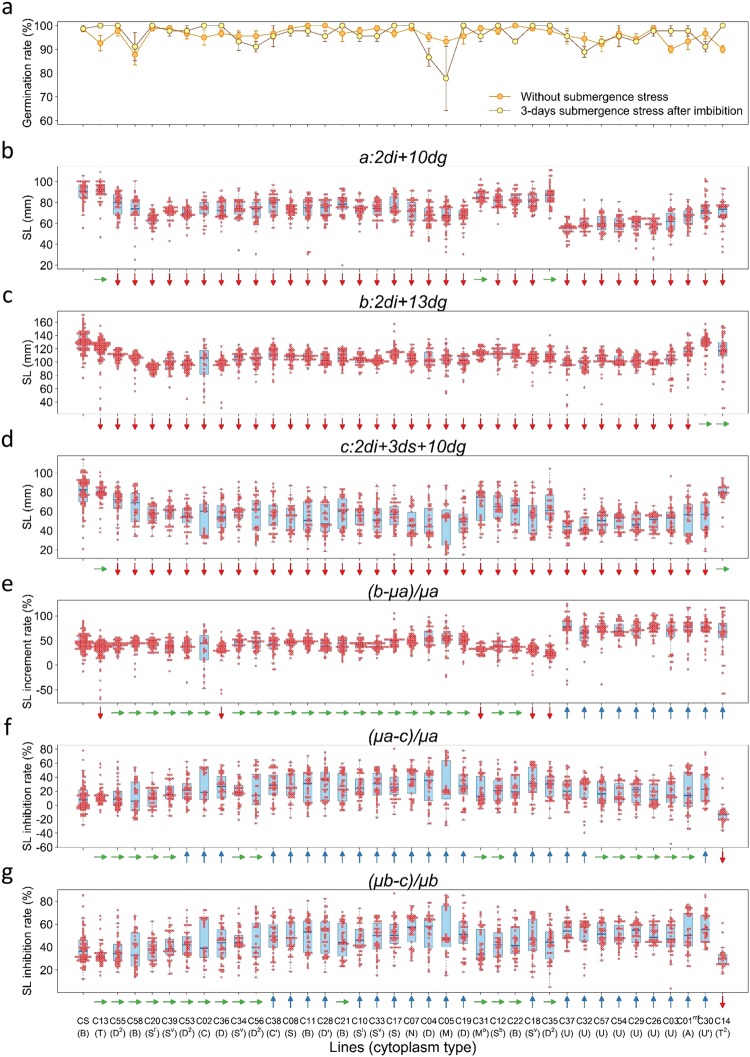
Germination and seedling growth with and without submergence among NC hybrid lines and the nuclear donor CS. (**a**) Germination rate, (**b**) shoot length (SL) at the 10^th^ day without submergence, (**c**) SL at the 13^th^ day without submergence, (**d**) SL at the 10^th^ day after 3 days of submergence, (**e**) SL increment rate (%) between the 10^th^ and the 13^th^ day without submergence, (**f**) SL Inhibition rate (%) by 3-days submergence as compared with variable *μa*, and (**g**) SL inhibition rate (%) by 3-days submergence as compared with *μb*. Fifteen seeds were used for each line and each condition and experiments were repeated three times, except for CS that was repeated six times (for variables *b* and *c*), C05 four times (for variable *a*), C02 (for variables *a* and *b*) and C55 (for variable *a*) twice. Pair-wise comparisons between CS and NC lines were made by Steel test. Sideway, downward and upward arrows respectively indicate no differences, significant decreases and increases in variables as compared with CS at a significance level of <0.05. For results of multiple rank sum comparisons by Steel-Dwass test made among CS and NC lines and among all lines including hexaploidy wheat lines, see Supplementary Tables S1–S4.

In all three experimental schemes, germination rates were considerably high in all lines, irrespective of submergence treatment (Fig. 3a). Germination rates without submergence ranged from 80 to 100% with a grand mean of 96.3% in NC hybrids and CS, whereas those with submergence ranged from 78 to 100% with a grand mean of 96.1%. Pairwise comparison of the means with and without submergence showed that submergence imposed on imbibed seeds for 3 days did not significantly affect germination of NC hybrids under the experimental conditions.

**Figure 4:**
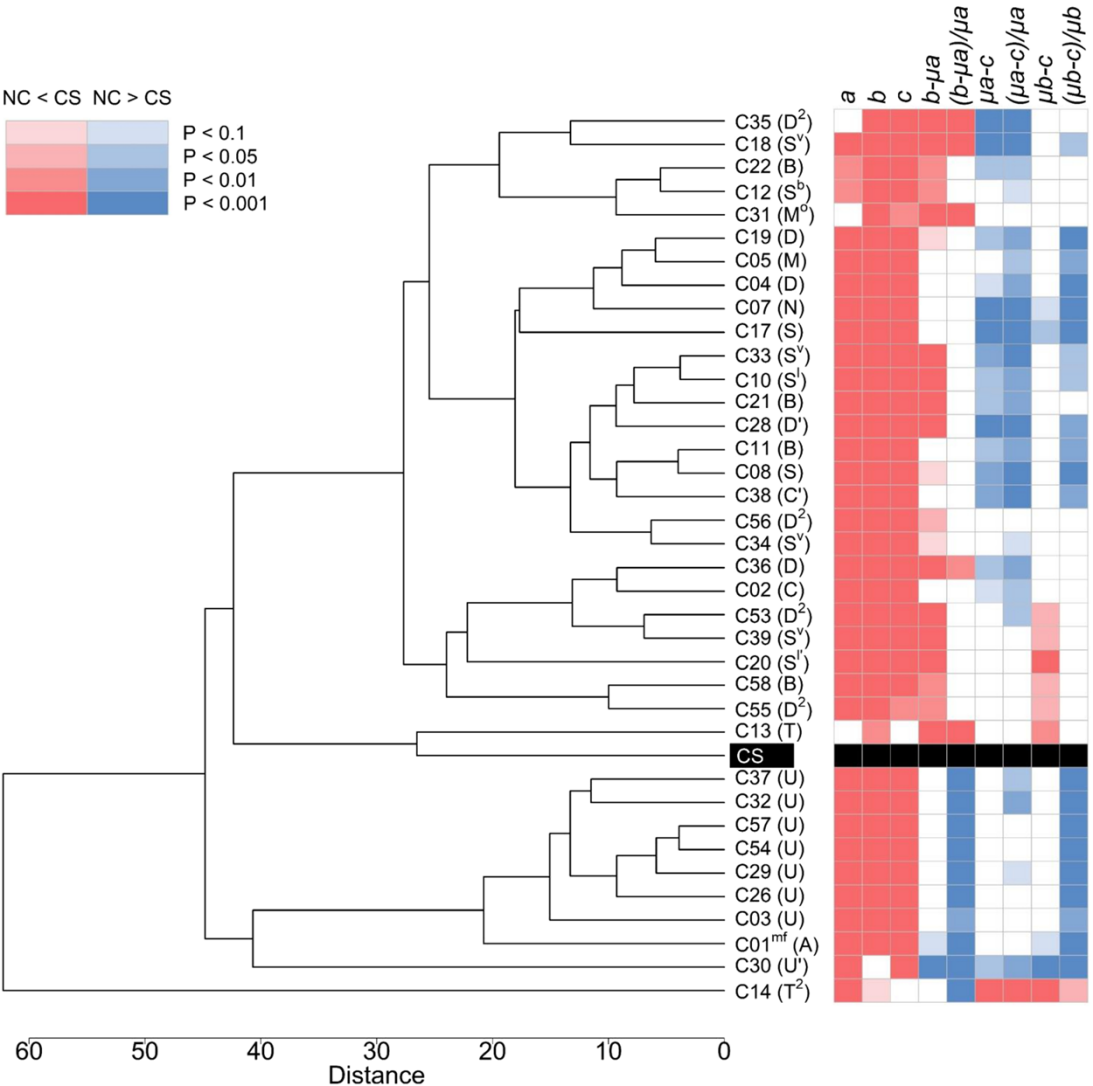
Cluster analysis of NC hybrid lines and the nuclear donor CS. Results of cluster analysis of NC lines and CS that were indexed by the nine variables of shoot length as depicted in Fig. 2. Hierarchical clustering by UPGMA was performed based on distance matrix calculated by Euclidean method. A heat map on the right showing significance levels of differences were made according to Steel test. Red and blue boxes respectively indicate smaller and greater values in NC hybrids compared with CS at the significance levels shown in the upper left. Smaller values of growth inhibition measured by variables *μa-c* and *μb-c*, and inhibition rate by variables (*μa-c*)*/μa* and (*μb-c*)/*μb* indicate higher levels of tolerance.

Seedling growth measured by five traits gave similar results overall, and hence we presented only shoot length (SL) data in the following analyses (for the other four traits, see Supplementary Figs S1 to S3). At the 10^th^ day of incubation, a majority of NC hybrids showed smaller values of variable *a*, except for C13, C31 and C35, all of which showed equivalent values compared with CS (Figs 3b, 4; Supplementary Fig. S1). Particularly, all NC hybrids (C03, C26, C29, C30, C32, C37, C54 and C57) possessing U and U′ cytoplasms showed marked reduction in variable *a* than CS and the other NC lines. At the 13^th^ day of incubation, C14 and C30 showed equivalent levels of variable *b* to that of CS, but all others showed significantly smaller values (Figs 3c and 4; Supplementary Fig. S2). With submergence, variable *c* significantly decreased in all NC hybrids except for C13 and C14, both of which showed no significant differences compared with CS (Figs 3, 4; Supplementary Fig. S3). Photos of representative seedlings of CS, C14 and C26 were shown for visual comparisons of seedling growth depicted by variables *a, b* and *c* (Fig. 2b–d).

We further studied effects of cytoplasmic substitution on seedling growth using six other variables, which were derived from the three measured variables. Magnitude of SL increment (*b-μa*) between the 10^th^ and 13^th^ day was larger in C01^mf^ and C30 than CS and other NC hybrids (Fig. 4; Supplementary Fig. S4). Relative rate of SL increment estimated by variable *(b-μa)/μa* between the 10^th^ and 13^th^ day were significantly larger in C01^mf^, C14 and all eight NC hybrids possessing either U or U’ cytoplasms than in CS and other NC hybrids (Figs 3e, 4). This was likely due to their smaller growth increment of *a* (Fig. 3b), which was pronounced at the earlier stage of seedling growth. Magnitude of SL inhibition evaluated by variable *μa-c* and relative rate of SL inhibition evaluated by *(μa-c)/μa* were greater in many NC hybrids than in CS, but in C14 both were smaller (Figs 3f, 4; Supplementary Fig. S4). C14 also showed a smaller SL inhibition and inhibition rate evaluated by *μb-c* and (*μb-c*)*/μb*, respectively (Figs 3g, 4; Supplementary Fig. S4). It was noted that C13, C20, C39, C53, C55 and C58 showed lower magnitudes of inhibition than CS when evaluated by variable µb-c (Fig. 4; Supplementary Fig. S4). The results suggested that recovery of shoot growth during de-submergence was greater in these NC hybrids than the other NC hybrids and the nuclear donor CS, particularly C14. On the other hand, all NC hybrids possessing U and U’ cytoplasms showed the greater inhibition rate than CS and the other NC hybrids. The results suggested that U and its derivative U’ cytoplasm exerted negative effects on submergence stress response. Statistical results of pair-wise comparisons between CS and NC hybrids by Steel test were shown in Fig. 3, and those of multiple rank sum comparisons among them by Steel-Dwass test were shown in Supplementary Tables S1–S4. Marked variabilities were evident among all lines, showing significant inter- and intraspecific diversity of cytoplasms. Two-way analysis of variance test confirmed significant interaction between cytoplasm and submergence stress (Table S5).

### Classification of cytoplasms based on submergence stress response

Cluster analysis was performed of 37 NC hybrids and the nuclear donor CS using the nine variables. A phylogenetic tree constructed by UPGMA (Unweighted Pair Group Method with Arithmetic mean) showed several unique features (Fig. 4). NC hybrid of C14, showing increased levels of submergence tolerance compared with CS and other NC hybrids based on all criteria in the bioassay, was an outlier in the dendrogram. All NC hybrids possessing U cytoplasms, which were characterized by their larger SL increment rates without submergence, formed a single cluster. C30 showing a greater magnitude and rate of SL growth increment without submergence formed a single cluster next to the cluster consisting of U and A cytoplasms. C13, which showed a smaller inhibition of SL after submergence stress than the other NC lines, was clustered together with CS. Five other NC hybrids, C20, C39, C53, C55 and C58 showing similar characteristics, were clustered in juxtaposition.

### Nuclear diversity assessed by seedling growth with and without submergence stress

**Figure 5:**
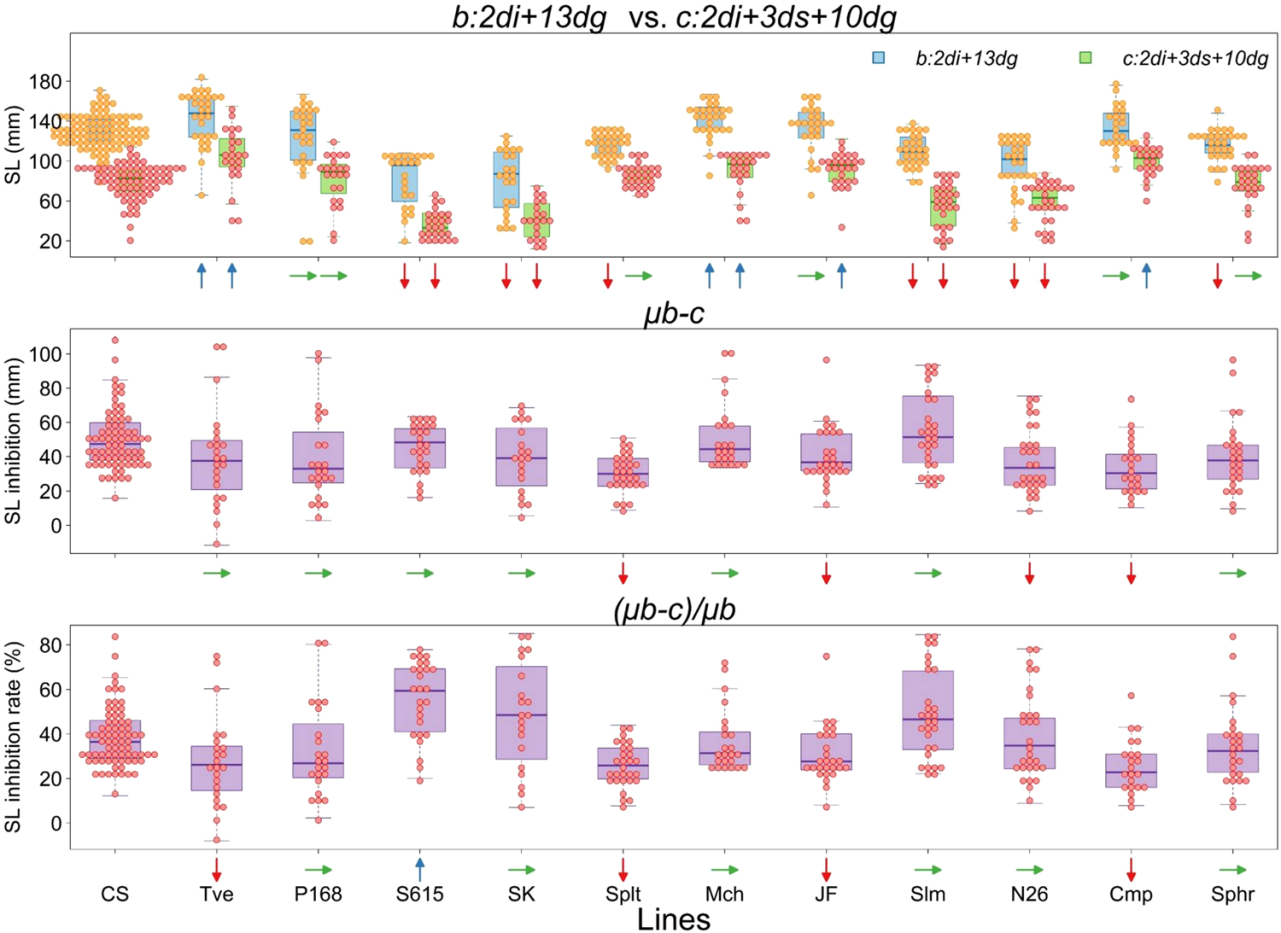
Seedling growth with and without submergence stress among 12 hexaploid wheat lines. (**a**) Comparison of shoot length (SL) measured by variable *b* (2di + 13dg) and *c* (2di + 3ds + 10dg) without and with submergence, respectively. (**b**) SL inhibition and (**c**) inhibition rate as estimated by variable *μb-c* and (*μb-c*)/*μb*, respectively, and expressed in negative values. Pair-wise comparisons between CS and NC lines were made by Steel test. Sideway, downward and upward arrows respectively indicate no differences, significant decreases and increases compared with CS at a significance level of <0.05. Smaller values of these variables indicate higher levels of tolerance. For results of multiple rank sum comparisons among all lines including NC lines, see Supplementary Tables S1–S4.

To compare magnitude of the observed cytoplasmic diversity affecting submergence response with that of nuclear diversity, we studied seedling growth of a collection of 12 hexaploid wheat lines (Table 1), which covered wheat genetic stocks with a wide range of morphological and geographical diversity^8,10^. Two variable *b* and *c* were measured, and their sensitivity to submergence was evaluated based on two derivative variables *μb-c* and (*μb-c*)/*μb*. A large variability existed among these euplasmic wheat lines (Fig. 5; see Supplementary Tables S3 and S4 for multiple rank sum comparisons among all lines including NC hybrids). Four lines, *T. aestivum* var. *erythrospermum* (Tve) that was used to make the first NC hybrid^6^, red winter wheat variety Jones Fife (JF), solid-stemmed spring variety S615, and hulled and non-free threshing *T. spelta* (Splt), showed significantly smaller values of both variables, indicating the higher submergence tolerance than CS. We then compared coefficients of variation (CV) among the hexaploid wheat lines and those of NC hybrids using these two variables, *μb-c* and (*μb-c*)/*μb* (Table 2). CV varied among NC hybrids and the hexaploid wheat lines, and an overall value of the latter was greater than that among the NC hybrids. CV among the NC hybrids, however, amounted as large as 63 and 67% of those among the wheat lines, suggesting a considerable level of cytoplasmic diversity in the submergence response.

**Table 2:** Statistical comparison of coefficient of variations within and between 37 NC hybrids and 12 hexaploid wheat lines.

variable	n	*μb-c*				*(μb-c)/μb*			
		Ave.	Var.	S.D.	C.V.	Ave.	Var.	S.D.	C.V.
NC hybrid lines	1,643	49.2	265.9	16.3	0.331	46.4	226.3	15.0	0.324
Hexaploid wheat lines	306	43.1	443.2	21.1	0.489	37.6	368.4	19.2	0.511

### Comparison of superoxide dismutase (SOD) activity in the nuclear donor CS and three NC lines with contrasting levels of submergence tolerance

**Figure 6:**
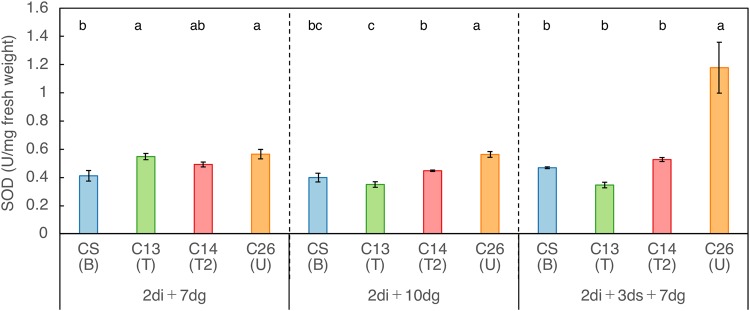
SOD activity in seedlings of the nuclear donor CS and three NC lines showing contrasting levels of submergence tolerance. SOD activities were measured after 2-days imbibition in seedlings grown for 7 and 10 days without submergence (2di + 7dg and 2di + 10dg, respectively) and seedlings grown for 7 days after 3-days submergence (2di + 3ds + 7dg). Significance of mean differences in SOD activity (Unit/mg fresh weight of whole seedling) among the lines were made by Tukey’ test at the 5% level.

Total SOD activity was measured using seedlings of CS and three NC hybrids (C13, C14 and C26), which showed contrasting levels of submergence tolerance/susceptibility. Triplicate samples of whole seedlings each grown in three different incubation conditions (2di + 7dg, 2di + 10dg, and 2di + 3ds + 7dg) were used. When compared among seedling emerged and grown under the same incubation conditions without submergence (2di + 7dg and 2di + 10dg), SOD activity showed some significant differences among all lines (Fig. 6). In contrast, with submergence ((2di + 3ds + 7dsg), a marked increase occurred in C26, which was highly susceptible to submergence stress (Figs 2d–g, 4; Supplementary Fig. S4).

## Discussion

Imbibition and germination are two important stages of developmental phase transition from quiescence state to vegetative growth in plants^44^. Normal and rapid process of this transition can be disturbed under unfavorable environmental conditions, causing low and uneven germination with less vigorous seedling growth, which often results in failure of good crop establishment needed for maximum productivity^45^. We examined involvement of cytoplasmic genomes and their diversity in the control of submergence stress response in wheat, which is prerequisite to study nucleus-cytoplasm interaction regulating submergence response. We used 37 lines of NC hybrids, in which distinct cytoplasms of the *Triticum-Aegilops* complex were combined with a common nucleus of wheat cultivar CS. Adopting the experimental schemes, which were suitable for evaluating submergence response without significantly affecting seed germination (Figs 1, 2), we studied seedling growth during de-submergence after subjecting imbibed seeds to the stress. A majority of NC hybrids possessing heterologous cytoplasms exhibited greater degrees of shoot growth inhibition compared with the nuclear donor after submergence. C14 with T^2^ cytoplasm derived from *Ae. mutica* showed a significantly smaller magnitude of inhibition and a relative inhibition rate than the nuclear donor CS and the other NC hybrids as judged by all variables used for the assessment of submergence response (Figs 3, 4). T^2^ cytoplasm gave no adverse effects on seedling growth of C14 both with and without submergence stress (Figs 2b, 3). It was also noted that NC hybrids of C13 with T cytoplasm of *Ae. mutica*, C20 with Sl’ cytoplasm of *Ae. longissima*, C39 with S^v^ cytoplasm of *Ae. kotchyi*, C53 and C55 with D^2^ cytoplasm of *Ae. juvenalis* and *Ae. crassa 6x and* C58 with B cytoplasm of *T. aestivum* ssp. *tibetanum* showed less growth inhibition when evaluated by variable *μb-c* (Fig. 4; Supplementary Fig. S4). Our results suggest that cytoplasms of these NC hybrids, particularly T^2^ cytoplasm of *Ae. mutica*, have potential to improve submergence tolerance, at least in the nuclear background of CS. By contrast, U and U’ cytoplasms of *Ae. umbellulata* (C03), *Ae. triuncialis* (C26), *Ae. biuncialis* (C29, C37), *Ae. triaristata* (C32, C54, C57) and *Ae. columnaris* (C30) caused greater rates of growth inhibition in all NC hybrids carrying them (Figs 3g, 4).

*Ae. mutica* is an annual and outcrossing diploid grass species growing endemically in the Anatolian Plateau in Turkey, Armenia and the northwestern part of Iran. This wild relative of wheat possesses two distinctly differentiated cytoplasms, *i.e*. T and T^2^. T^2^ cytoplasm causes almost complete and universal male sterility in all tested cultivars of common wheat including non-free-threshing *T. spelta* and *T. macha* without no other adverse effects than a delay of heading^46,47^. Restriction fragment length polymorphism analyses of mitochondrial DNA showed a small but clear differentiation between the two cytoplasms^48^. C02 with C cytoplasm of *Ae. caudata* and C05 with M cytoplasm of *Ae. comosa* used in our study also are male-sterility-inducing cytoplasms (Table 1), suggesting that male sterility was not directly related to submergence stress response. Habitats of *Ae. mutica* with T and T^2^ cytoplasms are overlapped mainly on slopes above irrigation ditches along the roads and abandoned or fallow fields, thus likely subjected to seasonal disturbances by irrigation water or rainfall in winter^49^. This might have some relation to submergence response exhibited by the NC hybrids with the cytoplasms of *Ae. mutica*. Although a reason for the observed differences in their response to submergence remains unknown, T^2^ cytoplasm might give advantageous effects over T cytoplasm through its interaction with a nucleus of CS. Further study is needed to test this possibility.

Phylogenetic tree of 37 NC hybrids and their nuclear donor CS was constructed by the UPGMA clustering based on all the nine variables (Fig. 4). Not only the cytoplasms derived from the different species but also the same cytoplasm types or subtypes derived from the same species showed significant differences in submergence stress response, revealing both interspecific and intraspecific diversity among the cytoplasms of the *Triticum-Aegilops* complex. The tree also showed some clear differences as compared with the previous reports based on phenotypic traits of 46 NC hybrids with 12 different nuclear genomes and their mitochondrial and chloroplast DNA polymorphisms detected by RFLP analyses. T^2^ cytoplasm was an outlier, and U cytoplasm formed a juxtaposed cluster that was distantly related to T and U’ cytoplasm in the dendrogram constructed based on the submergence response. This agreed with the results obtained by a number of phenotypic traits, in which T^2^ cytoplasm was distantly related to T, U and U’ cytoplasms^10^. Mitochondrial DNA polymorphisms also showed that both T^2^ and T cytoplasms were distantly related to U and U’ cytoplasms^48^. On the other hand, based on chloroplast DNA polymorphisms, a cluster of T^2^ and T cytoplasms were in close proximity to a cluster of U cytoplasm^50^. Similarity estimation of cytoplasms thus differed depending on different criteria used. Nevertheless, our phenotypic assessment of submergence stress response and clustering analysis suggest the uniqueness of T^2^, T and U and its derivative U’ cytoplasms in the *Triticum*-*Aegilops* complex.

As discussed above, marked diversity was evident among heterologous cytoplasms affecting submergence stress response in the nuclear background of CS. An important question was how large or small was the cytoplasmic contribution compared to the nuclear contribution. In wheat, much more efforts have been devoted to study waterlogging than submergence^32,33^. A clear genetic diversity was reported to exist in waterlogging tolerance^34^. For submergence, bread wheat was reported more tolerant than durum wheat and barley^37^. We studied nuclear diversity using 12 wheat lines covering a wide range of morphological and geographical diversity^8^. A large variability was observed among these hexaploid wheat lines (Fig. 5). Furthermore, the comparison of overall coefficients of variation among them and NC hybrids showed that cytoplasmic diversity observed in the nuclear background of CS was as large as over 60% that of nuclear diversity (Table 2). The observed difference suggested divergent response in NC hybrids that might be due to cytoplasmic differences exerted through nucleus-cytoplasm interactions.

Various mechanisms that control metabolism, growth, morphological characteristics and gene expression under submergence have been reviewed^51^. Submergence is known to limit oxygen diffusion into plant cells and tissues causing hypoxic damage, which is mediated by the production of reactive oxygen species (ROS)^35,36,52^. De-submergence is also detrimental because oxidative stress disturbs physiological processes producing ROS upon exposure to aerial oxygen^42^. It is crucial to understand how heterologous cytoplasms regulate submergence stress response. Therefore, as the first step, we studied the activity of superoxide dismutase (SOD), which acts in the conversion of superoxide radicals to molecular oxygen and hydrogen peroxide. SOD is composed of metalloenzymes Fe-SOD localized in chloroplasts, Mn-SOD in mitochondria and peroxisomes, and Cu/Zn-SOD in many cellular compartments including the three organelles. We measured a total SOD activity by adopting experimental schemes, in which effects of hypoxic stress on imbibed seeds during prolonged submergence and possible oxidative stress upon and during de-submergence could simultaneously be studied (Fig. 2). Comparisons of the activity among CS and three NC hybrids showing contrasting levels of submergence tolerance revealed a marked increase of SOD activity in C26 with U cytoplasm of *Ae. triuncialis* (Fig. 6), which showed a higher susceptibility than the others (Figs 3, 4).

Redox homeostasis is maintained by the balance between the production and scavenging of ROS. Among many enzymatic ROS scavenging systems, SOD is known as a major defense factor against ROS produced and accumulated under various environmental stresses in plants including wheat^53–56^. We measured the total activity of SOD in seedlings grown for 7 days after subjecting imbibed seeds to submergence for 3 days. We observed an elevated SOD activity in seedlings of the susceptible NC line C26 in comparison with the nuclear donor CS and the tolerant lines C14 and C13 (Fig. 6). The result seemed to conflict with reports showing that stress tolerant genotypes tended to maintain higher levels of antioxidative capacity^54^. It has also been known that SOD activity and ROS level do not necessarily show a positive correlation^57–59^. Nevertheless, our observation is notable in that the elevated SOD activity was accompanied by the growth retardation in the susceptible seedlings. Germination started, as judged by the elongation of coleoptile length longer than 15 mm, at around the 4th day of incubation of submerged seeds in the nuclear donor and the tolerant lines, but germination in the susceptible line was delayed, leading to the retarded seedling growth. This suggested that high levels of oxidative stress due to elevated ROS levels were remained in the susceptible seedlings that emerged from the submerged seeds. Production and detoxification of ROS is affected by various factors including the duration and intensity of applied stresses, tissue types and developmental stages. To clarify the relationship between these two complex processes regulating submergence stress response, it is necessary to follow the detailed time course of production, accumulation and detoxification of ROS at the early stages of seedling emergence and growth.

Taken together, we posit that significant diversity affecting submergence response exists among different cytoplasms of the *Triticum-Aegilops* complex. In plants, various adaptive traits including abiotic stress responses are predicted to be under control of nucleus-cytoplasms interaction, both through anterograde and retrograde regulation, in the latter of which various signaling factors generated by cytoplasmic organellar genomes can regulate gene expression of target nuclear genomes^30,31,59,60^. Further comparative studies of NC hybrids with different nuclear backgrounds are needed to clarify molecular mechanisms explaining how cytoplasmic genomes can regulate the submergence stress response through the interaction with nuclear genomes in wheat.

## Materials and Methods

### Plant materials

Thirty-seven NC hybrids and 12 euplasmic wheat lines were used (Table 1). NC hybrids possessed 24 distinct cytoplasmic genomes derived from four *Triticum* and 24 *Aegilops* species, which were individually combined with a nuclear genome of CS. Seeds of original NC hybrid lines produced by K. Tsunewaki^8,10^ were provided to C. Nakamura in 1988. Self-fertile lines and male-sterile lines have since been backcrossed and/or self-fertilized, and seeds harvested in July 2016 were used throughout the study. Wheat lines used included 12 representative strains from five diverse hexaploid wheat species including *T. aestivum, T. sphaerococcum, T. compactum, T. spelta and T. mach*, which were provided by S. Nasuda, Kyoto University, National Bio-Resources Project, in 2016. These lines were multiplied once and seeds harvested in July 2017 were used.

### Bioassay methods for studying sensitivity of seedlings and imbibed seeds to submergence stress

Test tube bioassay developed and successfully used for QTL mapping of submergence tolerance and analysis of gene expression associated with submergence in rice^43,61,62^ was adopted with modifications. For time-course study, seeds of CS (10 seeds at each time point) were imbibed individually for one day in test tubes (inner diameter of 14 mm, height of 165 mm) filled with 15 ml of deionized water (10 cm in depth). Water was drained out at indicated time points, and imbibed seeds were kept with embryo side up and incubated for up to 17 days. Care was taken to maintain an adequate amount of water by pipetting every day or every other day. Sensitivity of seedlings was studied by adding 15 ml of deionized water to cover growing seedlings at indicated time points and keeping them submerged for additional 7 days. Sensitivity of imbibed seeds was also studied by immersing 15 seeds at each time point continuously for up to 11 days followed by incubation under de-submergence conditions for additional 10 days for germination and subsequent seedling growth. Incubation conditions of seeds and seedlings were adjusted at day/night temperatures of 15 °C/10 °C with a photoperiod of 12hL/12hD under LED lumps at a light-intensity of ca. 120 μmol m^−2^ s^−1^ in a walk-in incubator. These temperature conditions were close to those in November when wheat seedlings were at the early stage of growth in the Kansai area of Japan. Germination was judged based on protrusion of a radicle with two seminal roots and a coleoptile length longer than 15 mm. Seedling growth was assessed by shoot length and fresh weight, root length and fresh weight, and total seedling fresh weight.

### Experimental schemes for studying cytoplasmic and nuclear diversities affecting submergence response of imbibed seeds

Seeds of 37 NC hybrid lines and 12 wheat lines (Table 1) were imbibed for 2 days, and then incubated under non-submergence conditions for allowing imbibed seeds to germinate and emerged seedlings to grow. Incubation conditions of seeds and seedlings were the same as described. Three experimental schemes were adopted for assessing the sensitivity to submergence stress as depicted in Fig. 2. Submergence stress was imposed on imbibed seeds by keeping them immersed for additional 3 days, and after releasing the stress they were incubated for 10 days under de-submergence conditions (2di + 3ds + 10dg). Imbibed seeds were also incubated for 10 and 13 days without stress (2di + 10dg and 2di + 13dg, respectively). Three variables *a, b* and *c* and six derivative variables measured based on the five traits were used for assessing seedling growth. Fifteen seeds each of wheat lines and NC hybrid lines were subjected to each experiment, and all experiments were repeated three times, except for CS that was repeated six times (for variable *b* and *c*), C05 four times (for variable *a*), C02 twice (for variables *a* and *b*), and C55 twice (for variable *a*).

### Statistical analyses of phenotypic data

Pair-wise comparisons between CS and NC lines were made by Steel test, and multiple rank sum comparisons among all NC hybrids and hexaploid wheat lines including CS were made by Steel-Dwass test, both using a software “EZR”^63^. Magnitude of variabilities within and between NC hybrids and wheat lines were compared based on coefficients of variations (CV). Distance matrix was calculated based on Euclidean method using the nine variables, and clustering by Unweighted Pair Group Method with Arithmetic mean (UPGMA) was performed using R package “fastcluster”^64^.

### Measurement of SOD activity

Activity of total superoxide dismutase (SOD) was measured using a nuclear donor CS and three NC lines of C13, C14 and C26 showing contrasting levels of submergence response. Seedings grown for 7 days after 3-days submergence (2di + 3ds + 7dg) and ones grown for 7 and 10 days without submergence (2di + 7dg and 2di + 10dg) were used for the measurements. Whole seedlings were frozen with liquid nitrogen, ground using TissueLyser II (Qiagen) and suspended in 0.1 M phosphate buffer saline (pH 7.4). Measurements of SOD activity (Unit/mg fresh weight of whole seedlings) were made in triplicate samples using SOD Assay Kit-WST (Kagaku-Doujin, Kumamoto, Japan). SOD activity was defined as the amount of enzyme that inhibited the production of soluble formazan from tetrazolium salt WST-1 by 50% according to the supplier’s instruction. Mean comparison among the lines was made by Tukey’s test. a, b, c in the upper corner indicate significant difference at the 1% level.

## Electronic supplementary material

Figure S1, S2, S3 and S4. Table S1, S2, S3, S4 and S5

## Data Availability

All data generated or analyzed during this study are included in this published article (and its Supplementary Information files).
