# Supplementary material for: Genetic diversity of submergence stress response in cytoplasms of the Triticum-Aegilops complex
Source: Sci Rep. 2018 Nov 2;8:16267. doi: 10.1038/s41598-018-34682-3 (PMC6214928; doi:10.1038/s41598-018-34682-3)
Supplement: Supplementary file 1 — Figure S1, S2, S3 and S4. Table S1, S2, S3, S4 and S5 [file 41598_2018_34682_MOESM1_ESM.docx]

**Supplementary Information**

**Genetic diversity of submergence stress response in cytoplasms of the *Triticum-Aegilops* complex**

**S. Takenaka, R. Yamamoto and C. Nakamura**

**Supplementary Figures**

**Figure S1.** Variable *a* as assessed by (**a**) shoot fresh weight, (**b**) root length, (**c**) root fresh weight and (**d**) total seedling fresh weight.

**Figure S2.** Variable *b* as assessed by (a) shoot fresh weight, (**b**) root length, (**c**) root fresh weight and (**d**) total seedling fresh weight.

**Figure S3.** Variable *c* as assessed by (a) shoot fresh weight, (**b**) root length, (**c**) root fresh weight and (**d**) total seedling fresh weight.

**Figure S4.** Derivative variables (**a**) *b-μa*, (**b**) *μa-c* and (**c**) *μb-c* as assessed by shoot length.

**Supplementary Tables**

**Table S1. Steel-Dwass test among NC hybrids based on variables *b* and *c*.**

**Table S2. Steel-Dwass test among NC hybrids based on variables *μb-c* and (*μb-c*)*/μb.***

***.***

**Table S3. Steel-Dwass test among NC hybrids and hexaploidy wheat lines based on variables *b* and *c.***

**Table S4. Steel-Dwass test among NC hybrids and hexaploidy wheat lines based on variables *μb-c* and (*μb-c*)*/μb.***

**Table S5. Two-way ANOVA test for estimating significance of submergence stress, cytoplasms and their interaction based on shoot length using the variables *a* and *c*.**

Supplementary Figure S1

Supplementary Figure 2

Supplementary Figure S3

Supplementary Figure S4

Supplementary Table S1

Supplementary Table 2

Supplementary Table 3

Supplementary Table 4

Supplementary Table 5
